# Satisfaction of health informatics professionals with Ethiopian health system: the case of three zones in Ethiopia

**DOI:** 10.1186/s12913-023-09623-0

**Published:** 2023-06-10

**Authors:** Girma Gilano, Sewunet Sako, Belachew Boranto, Firehiwot Haile, Hadiya Hassen

**Affiliations:** 1 Department of Health Informatics, School of Public Health, College of Medicine and Health Sciences, Arba Minch University, Arba Minch, Ethiopia; 2 Department of Pharmacy, School of Medicine, College of Medicine and Health Sciences, Arba Minch University, Arba Minch, Ethiopia

**Keywords:** Satisfaction, Health informatics, Health facilities, Ethiopia

## Abstract

**Background:**

The importance of the health information system faces multiple challenges such as supply, acceptance, and pressure from other professions in Ethiopia. Work-related challenges might cause low professional satisfaction and hinder service provision. There is a paucity of evidence for policy decisions to improve these challenges. Therefore, this study aims to assess Health Informatics professional satisfaction in the Ethiopian health system and associated factors to provide evidence for future improvements.

**Methods:**

We conducted an institutions-based cross-sectional study on health informatics professionals in three zones in Southern Ethiopia in 2020. We used a simple random sampling technique to select 215 participants. The local health officials were contacted regarding the research questions, and letters of permission were collected for data collection.

**Results:**

Out of 211(98%) Health Informatics professionals who accepted the interview, 50.8% (95%CI: 47.74%-53.86%) were satisfied. Age (AOR = 0.57; 95% CI: 0.53, 0.95), experience (AOR = 5; 95% CI: 1.50, 19.30), working time (AOR = 1.35; 95% CI: 1.10, 1.70), working as HMIS officers (AOR 2.30; 95% CI: 3.80, 13), single marital status (AOR = 9.60; 95% CI: 2.88, 32), and urban residence (AOR = 8.10; 95% CI: 2.95, 22) were some of the associated factors.

**Conclusions:**

We found low satisfaction among health informatics professionals compared to other studies. It was suggested that the responsible bodies must keep experienced professionals and reduce pressure from other professions through panel discussions. Work departments and working hours need consideration, as they are the determinants of satisfaction. Improving educational opportunities and career structure is the potential implication area.

## Background

Health Informatics programs are an essential tool that supports the health system. The Health system increasingly demands policy change, prioritization, resource allocation, monitoring of good impact, and progress of health programs using health information [1–4]. Decision-making in the health system depends on the timely collection, analysis, and dissemination of health information [5]. Health information is a facilitator of health system development and renovation of the health sector. Health information has been applied in designing, developing, planning, implementing, maintaining, and evaluating health systems [6].

The need for a health informatics program emerges from the relevance of the role of maintaining health records and medical services. However, many challenges hinder the achievement of program goals [3, 7]. These challenges include the lack of a national coordination system, an unsupportive health policy, a poor reflection on the need for Health Informatics, and the lack of planning and implementation commitments to support the Health Informatics profession [4, 6, 8]. Challenges are the potential cause of professionals’ dissatisfaction and can lead to poor achievement in Health Informatics-related goals, which can further disrupt patients’ satisfaction [8, 9].

Evidence from Botswana indicates that health decision-making faces many challenges. These challenges can be managed by validating and processing routinely collected health data by Health Informatics professionals [10]. Another study in South Africa underlined that the source of challenges to Health Informatics professionals are related to the profession's expectations and contribution to the health system. The dilemma is that pressure limits professional capacity and work interest, and authorities expect more performance [11]. Poor health professionals’ perception is associated with the data collection and poor culture of information use. This puts Health Information professionals and health information systems at a lower importance level compared to other health professions [12]. This means implementation of health information systems alone is not enough to solve health system problems regarding data completeness, timeliness, reliability, and accuracy unless environmental issues such as workplace pressure and poor perception of health professionals toward health information systems improved as outlined by Garrib et al. [13]. As a solution, a pilot program from Sierra Leone suggested that community engagement is the key to easy unnecessary pressure [14]. However, another study in South Africa indicated that the problem goes back to initial implementations. It indicated that authorities and other professionals understand health information systems after implementing all components and after the achievement of the overall change in management support [15]. A systematic review related to the implementation of the health information system showed that the system was implemented without a coordinating framework and had no strong leadership links in some departments [16]. For instance, Health Informatics professionals in Sub-Saharan countries mostly wanted to drop the system because of poor implementation strategies [17]. Overall, there are many challenges in Health informatics programs in Africa that might affect the satisfaction of professionals in the domain.

In developing countries, limited resources, poor job satisfaction in the health system, inadequate technology skills, and the inability of health systems to incorporate and support technological facilities impose further pressure on the Health Informatics profession [3, 18, 19]. A well-established health information system positively supports the function of the entire health system [20, 21].

In Africa, there is no evidence regarding Health Informatics professionals’ satisfaction. The existing evidence is highly focused on data utilization and Health Management Information System (HMIS) implementation [3, 22, 23]. Professional satisfaction is the key to the success of a given profession. Poor satisfaction with Health Informatics professionals can be a problem for clinical safety, security, information transfer, and knowledge sharing as they can cause ineffectiveness and failure of the entire health system [24, 25].

In Ethiopia, there was no study in the Health Informatics field until 2005 because the program was not started, and there were no training centers or teaching institutions in the country related to Health Informatics. Although some studies appeared after 2005, they focused on HMIS utilization and implementation [26, 27]. Additionally, the lack of knowledge about Health Informatics, the absence of occupational standards, and the lack of career development structure become additional challenges to Health Informatics [3, 28, 29]. Moreover, health professionals see health information systems as a burden to the already overburdened health system [30, 31]. The reason is mostly related to the time it takes for data collection and manipulation. This may negatively affect the satisfaction of Health Informatics professionals.

However, Ethiopia had an overall poor job satisfaction in many programs. For example, only 53.8% of health professionals were satisfied in Addis Ababa health centers [32]. Only 41.7% of health professionals were comfortable with their job in another study [33]. Other statistics show that health professionals’ satisfaction was 31.7% in the Western Amhara region [34], 46.68% in a systematic review and meta-analysis in Ethiopia, 54% in the University of Gondar referral hospital [35], 55.2% in Bahir Dar Hospital, and 46% in the West Shoa Oromia region [36]. This illustrates that overall health professionals’ satisfaction is not good in the country. It also indicates that there is limited evidence in the Health Informatics profession. The challenges might be worse for the newly started Health Informatics program. Thus, the current study aims to assess the Health Informatics professionals’ satisfaction and associated factors to provide information for policy decisions in Ethiopia.

### Conceptual framework

**Fig. 1:**
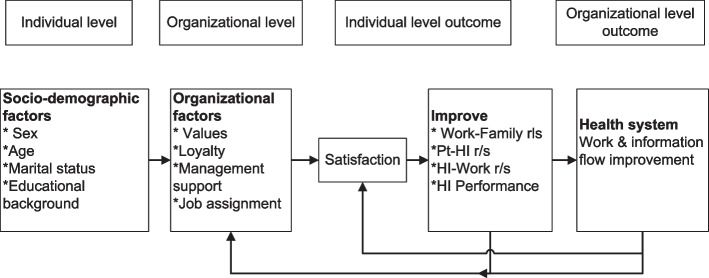
The conceptual framework depicting Health Informatics satisfaction from previous literature. NB: pt = patient; HI = Health Informatics; r/s = relationship

Figure 1 below illustrates the complex effect of satisfaction on Health Informatics professionals, which is adapted from previous literature. There are many ways to conceptualize satisfaction in literature, but Fig. 1 better explains our objectives [16, 17, 37, 38]. Health Informatics satisfaction is affected by both individual and organizational level factors. A satisfied Health Informatics professional can improve social, patient, and workplace relationships and performance. This is important to sustain work and information flow [37, 38]. The health system improvement is just showing direction and is not assessed here.

## Materials and methods

### Study setting

This study is conducted in the Southern Nations Nationalities and Peoples’ Regional (SNNPR) state, which covers 10% of the country’s total area & 20% of the country’s population. Gamo Gofa, South Omo, and Segen are the three zones included in this study [39]. The study population is all health informatics professionals working in public health institutions in the three zones.

### Study design and participants

We conducted an institution-based cross-sectional study design in the three zones in SNNPR from August 2019 to April.2020.

In the Ethiopian health system, Health Informatics was started as a diploma and master’s program until 2012. The University of Gondar is the first to start a bachelor’s program [39]. There are some degree holders during the study period while the rest are diplomas. Our study does not include Information Technology, Computer Science, and Information System professionals. The reason is all those programs existed many years ago and are well established, unlike the Health Informatics program. We included only three zones in the region since; they are the only research catchment areas of Arba Minch University.

### Inclusion and exclusion criteria

We included all Health Informatics professionals working in the selected public health institutions/facilities. However, those Health Informatics professionals working in the randomly selected public health institutions for less than six months were not included because of the possible lack of experience in the program. We also excluded Health Informatics transferred out to other health institutions and those not accessible because of chronic or severe illness.

### Sampling and sampling procedure

We used a single population proportion formula considering the proportion of participants with reasonable satisfaction as 50% and level of precision ά = 5% with 95% CI and margin of error Δ = 5%, which gave 384. Because only 401 professionals were in the area, the sample reduction formula gave us 196, and adding a 10% non-response rate became 215. The assumption of considering 50% to calculate the sample size of a single-proportion population in the absence of any evidence is supported by many statistical guidelines. To the authors' knowledge, no satisfaction study on Health Informatics professionals in Ethiopia and Africa. Considering proportion as 50% provides the maximum sample size that no other proportion can provide [40–44].

To obtain the 215 Health Informatics professionals, we collected reports on the number of Health Informatics professionals from zonal and woreda health departments. From that information, there is a rare condition when more than one Health Informatics professional works in the same health institution because the number of professionals is limited. Depending on this information, we randomly selected health institutions with Health Informatics professionals using health departments’ lists as a frame. In health institutions where more than one Health Informatics professional worked, we used a lottery method to pick one professional per health institution.

### Variables

The outcome variable was Health Informatics professional satisfaction, measured by eight Likert scale items.

Independent variables: were age, gender, marital status, educational background, professional status, occupation, years of service, type of institution, employment, administrative duties, department/unit, hours worked per week, night shift, income, and residence, health informatics client relationship factors, work-related stress, and family-work conflict.

### Measurements

#### Satisfaction (health informatics professional satisfaction)

To estimate Health Informatics professionals’ satisfaction with colleagues, the work itself, promotions, remunerations, environment, facility, current job, and supervisors, we used a five-point Likert’s scale (1—strongly disagree, 2—disagree, 3—undecided, 4—agree and 5—strongly agree) (9). The overall job satisfaction of Health Informatics professionals was calculated by taking the average scores of the eight items. A Health Informatics professional who scores ≥ 3.5 (1) is satisfied, and < 3.5 (0) is dissatisfied (9).

#### Professional status

We measured professional work status in institutions using manager/supervisor, team leader, professional member, and lower primary/trainee categories. Supervisor/manager/ is equivalent to the senior/deputy senior professional member. The team leader here is the member who is in a lower position than the manager is in the organizational structure. The professional member is the primary professional member. The member whose status is below primary professional is a trainee or certificate level.

#### Work stresses

The work-related stress questionnaire includes feeling great pressure from work, feeling a high level of tension from work, and having trouble falling asleep. We used a five-point Likert’s scale (1—strongly disagree, 2—disagree, 3—undecided, 4—agree, and 5—strongly agree) to measure the relationship between work-related stress of Health Informatics professionals and satisfaction.

#### Work-family conflict

The work-family conflict assessment includes eight items, which were evaluated using a five-point Likert scale (1—strongly disagree, 2—disagree, 3—undecided, 4—agree, and 5—strongly agree). Items one to three are time-based work-family conflict, items four to six are behavior-based work-family-conflict, and items seven to eight are strain based.

#### Health informatics–patient relationship

The Health Informatics–patient relationship has four items. We used five points Likert’s scale to measure the relationship between patients and its effect on job satisfaction (1—strongly respectful, 2—respectful, 3—undecided, 4—disrespectful, and 5_strongly disrespectful).

#### Organization-related factors

The relationship between the organization and its influence on Health Informatics professional satisfaction was measured using four items. We used five points on Likert’s scale (1—strongly respectful, 2—respectful, 3—undecided, 4—disrespectful, and 5_strongly disrespectful).

### Data quality control

We conducted a pretest of the questionnaire on 5% of non-selected health facilities to check the acceptability and consistency of the questions. In addition, we trained the data collectors and supervisors for two days on the overall data collection procedure. The supervisor checked the completeness of the questionnaires each day in the field.

### Data collection personnel and instruments

We used a structured standard questionnaire adapted from previous studies with slight modifications [3, 10, 11, 32, 33]. Because of these modifications, we checked the consistency of the questionnaire, which showed a Cronbach’s Alpha of 0.794. We used 10 first-degree Health informatics and nurse professionals for data collection from Arba Minch University and Arba Minch College of Health Sciences. The data collectors were trained and supervised by master’s degree Health Informatics professionals from the same University.

### Data processing and analysis

We cleaned, processed, entered the data into Epi Info, and analyzed using SPSS version 0.25. We applied binary logistic regression to examine the relationship between explanatory and response variables. We also considered the mean Variance Inflation Factor, and it became 2.59, which is in the acceptable range. We used a p-value of < 0.25 to include variables in the final model and a *p*-value of < 0.05 to declare the presence of associations. We presented the descriptive data, using numbers, mean, percent, and standard deviation but for inferential statistics, we used Adjusted Odds Ratio (AOR) with a 95% CI.

## Results

### Socio-demographic characteristics

Of the 215 participants who approached, 211(98%) responded to the interview. More than half of the participants (64.5%) were male and the mean age of participants was 25.82 ± 3.36. The average year of experience was 3.21 ± 1.70 with 44.71 ± 8.13 working hours per week. The mean salary of the study participants was 3158 ETB with a standard deviation of 758 ETB per month. Only 36% of the participants reported overtime work payments, while the rest were not allowed to earn extra time payments they worked. Thirty-two percent of the participants worked in health facilities without electricity, while only 10.9% worked as health information team leaders (Table 1).

**Table 1:** The sociodemographic characteristics of health informatics professionals in three zone of SNNPR health facilities, 2020

Variables	N (%)
**Sex**	
Male	136(64.50)
Female	75(35.50)
**Marital status**	
Single	93(44.10)
Married	118(55.90)
**Residence**	
Rural	90(42.70)
Urban	95(57.30)
**Department**	
HMIS officer	42(19.9)
M&E	25(11.8)
HMIS focal person	75(35.5)
Planning & Evaluation	23(10.9)
MRU officer	35(16.6)
ART	7(3.3)
ZHD	4(1.9)
**Presence night duty**	
Yes	135(64.00)
No	76(36.00)
**Type of institution**	
Hospital	48(22.70)
Health center	114(54.00)
Health Office	49(23.20)
**Professional status**	
Team leader	23(10.9)
Committee member	97(46.00)
Lower/primary/trainee	71(33.6)
Other unspecified	20(9.50)

N.B: *HMIS* Health Management Information System, *M&E* Monitoring and Evaluation, *MRU* Medical Record Unit, *ART* Anti-retroviral Therapy, *ZHD* Zonal Health Department

### Satisfaction of health informatics professionals

The overall mean Health Informatics professional satisfaction was 2.54 ± 1.05, which is 50.8% (95%CI: 47.74%-53.86%). Although all parameters scored low on Health Informatics professional satisfaction, a relatively good score was observed in the health facility, colleague, and supervisor items 2.94 ± 1.10, 2.93 ± 1.26, and 2.81 ± 1.00 respectively (Table 2).

**Table 2:** The satisfaction of Health Informatics professionals working in public health facilities in three zones, in 2020

Satisfaction parameters	Sum	Mean	Std. Dev	Satisfied %
I’m satisfied with my colleagues	618	2.93	1.27	58
I’m satisfied with my work itself	539	2.55	1.02	51
I’m satisfied with my promotions	448	2.11	1.09	42
I’m satisfied with my remunerations	373	1.77	0.80	35
I’m satisfied with my work environment	516	2.45	1.06	48
I’m satisfied with my facility	592	2.81	1.0	56
I’m satisfied with my current job	578	2.74	1.11	54
I’m satisfied with my superiors	621	2.94	1.10	58
Mean		2.54	1.05	50.8

**Fig. 2:**
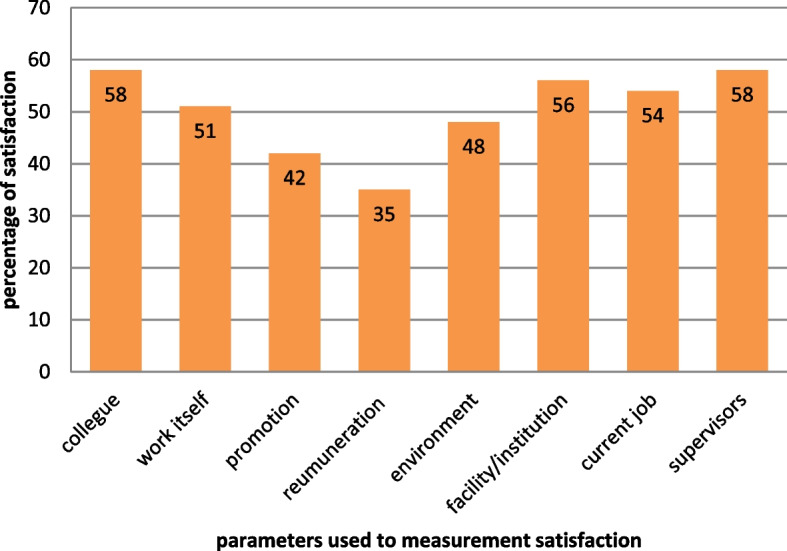
Distribution of satisfaction parameter percent among respondents in public health facilities in SNNPR, 2020

Promotion (42%), remuneration (35%), and work environment (48%) were items that scored low in Health Informatics professionals’ satisfaction (Fig. 2).

**Fig. 3:**
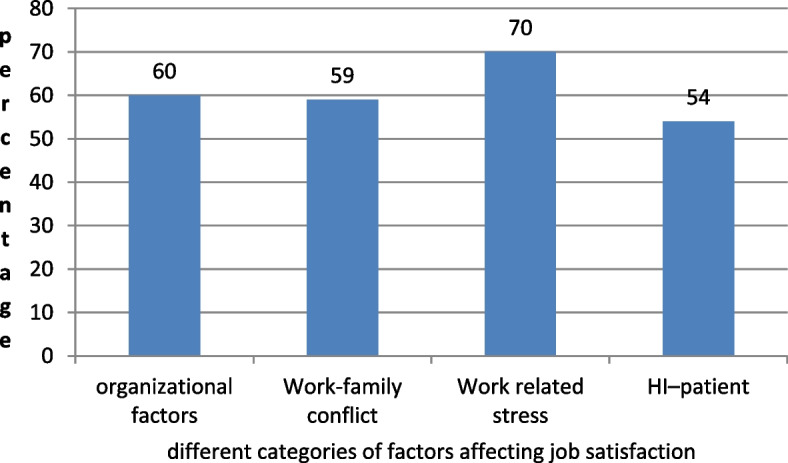
Distribution of different factor categories affecting Health Informatics professional satisfaction in three zone health facilities, 2020

More than half of Health Informatics professionals (54%) feel they have a good relationship with clients. Fifty-nine percent of them also reported family-work conflict while 70% of them feel high pressure from work (Fig. 3).

As age increased by one year, the odds of satisfaction was reduced by 43% among the participants (AOR = 0.57, 95%CI: 0.53, 0.95). Conversely, the odds of satisfaction increased as years of experience increased (AOR = 5, 95%CI: 1.50, 19.30). Additionally, the odds of satisfaction increased as working time increased (AOR = 1.35, 95%CI: 1.10, 1.70). Participants’ extra-time payments showed high odds of satisfaction (AOR = 1.90, 95%CI: 3.6, 7). The participants who work as HMIS focal personnel have high odds of satisfaction compared to HMIS officers (AOR = 2.30, 95%CI: 3.8, 13). Participants working in planning & development departments had higher odds of satisfaction AOR = 2.40, 95%CI: 2.20, 27). Married Health Informatics professionals were more satisfied than unmarried (AOR = 9.60, 95%CI: 2.88, 32), and rural residing participants have higher odds satisfaction compared to urban ones (AOR = 8.10, 95%CI: 2.95, 22) (Table 3).

**Table 3:** Distribution of factors associated with satisfaction of Health Informatics professionals in SNNPR public health facilities

Variables	*p*-value	AOR	Lower 95% CI	Upper 95% CI
Age	0.03	0.57	0.531	0.949
Department				
HMIS officer	1.00			
M&E	0.99	0.00	0.00	0.00
HMIS focal person	0.02	2.30	3.80	13.00
Planning & Evaluation	0.03	2.40	2.20	27.00
MRU officer	0.18	22.10	0.23	0.00
ART	1.00	0.00	0.00	0.00
ZHD	0.99	6.40	2.40	1.70
Marital status				
Single	1.00			
Married	0.01	9.60	2.88	32.00
Experiences	0.01	5.30	1.50	19.30
Original residence				
Urban	1.00			
Rural	0.01	8.10	2.95	22.00
Working hours per week	0.01	1.35	1.10	1.70
Night or other payment duty	0.01	1.90	3.60	7.90
Feel great pressure from work	0.01	2.45	1.22	4.90
Trouble falling asleep because of the work	0.00	0.33	0.18	0.60
Feel nervous because of the work	0.03	0.44	0.21	0.94
The degree to which patients respect You	0.03	8.6	2.10	35.45
Recent health informatics–patient relationship	0.03	2.03	1.04	3.94
Miss family activities due to work	0.01	0.02	0.01	0.50
Problem-solving behaviors make no sense at home	0.01	0.01	0.00	0.46
Behavior that is effective and necessary at work would be counterproductive at home	0.08	0.19	0.030	1.27
Drain prevents me from contributing to the family	0.04	0.03	0.003	0.34
Owing to the pressures from work, I do not want to do my favorite things at home	0.01	0.07	0.010	0.48
I feel very little loyalty to this organization	0.001	0.09	0.023	0.38
I would accept almost any type of job assignment to keep working for this organization	0.01	2.60	1.20	5.50
I am proud to tell others that I am part of this organization	0.004	5.50	1.70	17.98
The management of this organization is supportive of me	0.02	2.30	1.11	4.70

#### Work-stresses

Higher pressure from work was associated with higher odds of satisfaction (AOR = 2.45, 95%CI: 1.22, 4.9); however, professionals who get trouble sleeping and those who feel nervous because of work had 67% and 56% reduced odds of satisfaction (AOR = 0.33, 95%CI: 0.18, 0.60) and (AOR = 0.44, 95%CI: 0.2, 0.94) respectively.

#### Health informatics–patient relationship

The relationship between the professional and service takers indicated that ‘the degree of service taker respect’ and ‘good current relationship’ items showed higher odds of satisfaction (AOR = 8.6, 95%CI: 2.1, 35.45) and (AOR = 2, 95%CI: 1.04, 3.9) respectively.

#### Work-family conflict

The Health Informatics professionals that miss family things due to work had 98% reduced satisfaction (AOR = 0.02, 95%CI: 0. 01, 0.51). Similarly, items such as ‘problem-solving behaviors at home, ‘less contributing to the family due to work’, and ‘do not favor doing things at home for had 98.7%, 96.7%, & 93% reduced odds of satisfaction (AOR = 0.013, 95%CI: 0.00, 0.46), (AOR = 0.033, 0.003, 0.341), and (AOR = 0.07, 95%CI: 0.01, 0.487) respectively.

#### Organizational factors

The professionals who reported ‘less loyal’ had 90.6% reduced odds of satisfaction (AOR = 0.09, 95%CI: 0.23, 0.38). Additionally, items such as ‘accepting any type of duty’, ‘proud to tell being in this facility, and ‘supportive management’ items showed higher odds of satisfaction (AOR = 2.60, 95%CI: 1.20, 5.50), (AOR = 5.50, 95%CI: 1.70, 17.98), and (AOR = 2.30, 95%CI: 1.11, 4.70) respectively.

## Discussion

Health Informatics is a newly implemented program in the last two decades in Ethiopia. There has been many problem, which may hinder its establishment in the Ethiopian health system. This study aims to assess the satisfaction of Health Informatics professionals in Ethiopia’s health system. From our analysis, the overall satisfaction of Health Informatics professionals was 2.54 ± 1.05 or 50.8% (95%CI: 47.74%-53.86%). The finding is less than that of Wuhan Medical Informatics (58.28%) and consistent with Lahore health professionals’ satisfaction (53.6%) [45, 46]. The inconsistent information might indicate pressure on the Health Informatics profession in Ethiopian. It might also indicate the contextual differences. Remuneration (35%) and promotion (42%) are the two parameters that scored less on Health Informatics professionals’ satisfaction. Other evidence also shows that the two parameters have been the consistent source of dissatisfaction in the health system [21, 47]. Thus, remuneration and promotions are the key parameters that need change to improve Health Informatics professional satisfaction.

The satisfaction of Health Informatics professionals with their work was 51%. A report from another study is consistent with this finding showing that the work also contributes to dissatisfaction [45]. This shows that dissatisfaction is also attributable to overall low job satisfaction in Ethiopia [45, 46]. For instance, only 32% of health professionals were satisfied with their work in Ethiopia [48]. In this regard, some health professionals might have poor interest in their profession. In other words, supervision is key in the health system; though, only 58% of participants in our study were satisfied with their supervisors. This is lower than the 91.2% reported in China [47]. The inconsistency might be an indication of the difference in leadership style, setting, and development of Health Informatics.

Satisfaction decreased with the increased age of the participants. The inverse relationship between age and satisfaction is also reported by another related study in Western Ethiopia [49]. This might indicate that over time satisfaction among health professionals including Health Informatics decreases. Married participants showed higher satisfaction than single participants, which is also evident from other studies [24, 45]. At this point, we might link satisfaction to a stable family and social relationships.

Additionally, rural residents showed higher satisfaction than urban residents. This is also evidenced by higher job satisfaction in rural than urban areas from another study [50]. The pressure might be higher in urban areas and can be attributable to the low client or community-professional relationship [26, 45, 51].

The relationship between Health Informatics professionals and clients is positive during the analysis. Other studies also showed positive professional satisfaction with service-takers [21, 45]. This might show that the deteriorating factors are not community or client related, but might be due to the workplace, leadership, and pressure around the work. In the analysis, the negative relationship between families and Health Informatics is linked with poor satisfaction. Another study also reported an association of dissatisfaction with poor family-Health Informatics relationships [50], and it might be a potential link to future implications. Similarly, poor relationships between organizations and Health Informatics professionals were connected with poor satisfaction. A study in Pennsylvania showed that a positive relationship between organizations could improve employee satisfaction [36]. This might also indicate that the poor relationships between the profession, organization, and environment contribute to the dissatisfaction reported in this study [52].

However, the lack of implementation framework and strong leadership in Africa might be the source that creates poor links among Health Informatics professionals, health officials, the community, the organizations, and the work environment. Organizational culture, family, work itself, and social relationships might create further pressure that leads to dissatisfaction [16, 17]. Overall, our findings show that the effect of work stress, family-work-conflict, Health Informatics-community/patient relationship, and organizational environment might lead to low satisfaction. Furthermore, the initial source of challenges in the African context led to the lack of a strong national coordination system, an unsupportive policy system, the poorly substantiated importance of Health Informatics, and the lack of planning and implementation commitments in the countries and contributed to the problem [4, 6, 8].

The limitations of this study include a small sample size, the cross-sectional nature of data, and limited generalizability of the findings. The authors followed the scientific methods of determining the sample size. The findings should be used cautiously, considering the locality of the study. Recommendations were provided for wider area investigations and further implications that might be an awakening bell for Ethiopia and most African countries.

## Conclusions

The evidence shows that 50.8% satisfaction in our study is low compared to previous studies on health professionals. Promotions, remunerations, and the work environment are factors associated with decreased satisfaction. Evidence also shows that poor implementation and health leadership systems in Africa take a substantial portion in decreasing satisfaction. This shows that responsible bodies need to cover multidimensional approaches to solving the Health Informatics professionals’ multi factorial causes of poor satisfaction. Government commitments and strong workplace leadership to ensure equality among health professionals improve the challenges. Additionally, work experience, working hours, and work department should be an integral part of future implementations. We recommend quality studies to support future decisions regarding the Health Informatics profession.

## Data Availability

All the data generated or analyzed during this study are included in this article and will be available for the reason requested by the corresponding author.

## Abbreviations

AOR: Adjusted odds ratio
CI: Confidence intervals
HMIS: Health Management Information System
HI: Health Informatics
SNNPR: Southern Nation Nationality and Peoples Regional
